# Tuberculosis of the ear, a professional disease?

**DOI:** 10.1016/S1808-8694(15)30614-5

**Published:** 2015-10-18

**Authors:** Patrícia Maria Sens, Clemente I.R. Almeida, Lupércio O do Valle, Luís H.C. Costa, Miguel L.S. Angeli

**Affiliations:** 1 Master's degree student, physician.; 2 Doctoral degree, USP. Full professor, FMJ. Professor in the graduate course of the Faculdade de Ciências Médicas (Medical School), Santa Casa de Sao Paulo.; 3 Master's degree in communication disorders, PUC de São Paulo (Sao Paulo Pontifical Catholic University). Assistant physicians, Servidor Público Municipal Hospital.; 4 Master's degree in medicine, FCMSCSP. Assistant physician, FMJ.; 5 Master's degree student, FCMSCSP. Assistant physician, FMJ.

**Keywords:** occupational medicine, fungal external otitis, otitis media, tuberculosis

## Abstract

Tuberculosis is a rare cause of chronic suppurative otitis media and mastoiditis; the predisposing factors of this association, however, are not commonly described. There has been an alarming increase in the incidence of tuberculosis in Brazil, including tuberculous otitis media. These patients typically present multiple perforations of the tympanic membrane, an ear discharge, and progressive hearing loss. This diagnosis should be taken into account in patients that do not respond to routine therapy for fungal external otitis or bacterial otitis media. In this retrospective study, the authors describe four cases of patients with tuberculous otitis media. This sample consisted of two physicians, a chemical engineer and an underage child in whose family there were cases of active tuberculosis. Predisposing factors for tuberculous otitis were contact with family members that had tuberculosis, professional contact with patients and exposure to pathogenic microorganisms in airways.

## INTRODUCTION

Tuberculosis is a chronic bacterial infection caused by Mycobacterium tuberculosis, a slow-growing strict aerobic bacillus. It forms granulomas with caseous necrosis due to the cell response of involved tissues.^1^ Brazil is tenth worldwide in the incidence of tuberculosis, and the first in number of cases per year in the Americas; the disease affects 55/100,000 inhabitants, particularly in major urban centers. Certain Brazilian municipalities have even higher incidence rates, such as 210/100,000 (Cedro, PE), 100/100,000 (Manaus, AM) and 160/100,000 (Rio de Janeiro, RJ).^2^ A complicating factor in this disease, other than malnutrition, is the high treatment abandonment rate, making therapy successful in only 65% of cases; this situation merely disseminates contamination.^3^

Factors that have led to an increased incidence of tuberculosis in past decades include: the increased prevalence of HIV infection; an increased incidence of people living in poor social and economic conditions; increased resistance to anti-tuberculosis therapy; the development of resistant bacilli; drug dependency; diabetes; and alcoholism.^3^, ^4^, ^5^, ^6^, ^7^, ^8^

In the head and neck tuberculosis affects mostly the larynx, and to a lesser degree, the middle and external ear, the tonsils, neck lymph nodes, the pharynx, the mouth and salivary glands.^4^, ^7^, ^8^

In the beginning of the 20th century, tuberculosis caused 3% to 5% of chronic suppurative otitis media cases; this rate decreased with the advent of anti-tuberculosis therapy.^8^, ^9^ Its current frequency in developed countries ranges from 0.05 to 0.9% of the causes of chronic middle ear infection.^4^ This decreased incidence has meant that tuberculous otitis media is currently misdiagnosed, particularly in underdeveloped countries. This is due to lack of constitutional symptoms and the fact that signs and symptoms are frequently indistinguishable from non-tuberculous chronic otitis media.^4^, ^5^, ^10^, ^11^

The purpose of this paper was to review the literature on this theme, to report four cases of tuberculous otitis media, and to describe the predisposing factors, clinical features, the diagnosis, the progression, and therapy.

## REVIEW OF THE LITERATURE AND THE DIFFERENTIAL DIAGNOSIS

Middle ear tuberculosis as a primary site is rare.^1^, ^3^, ^4^, ^9^ It classically affects children more often than adults.^1^, ^11^

Although the pathogenesis itself is still controversial, some theories explaining middle ear tuberculosis infection have been developed,^1^, ^3^, ^5^, ^11^, ^12^, ^13^ namely: hematogenic transmission from distant sites, for instance, in miliary tuberculosis; by direct extension from the nasopharynx through the auditory tube, with or with no primary pharyngeal infection, particularly in children in whom milk infected with Mycobacterium bovis enters directly into the middle ear by the auditory tube; by lymphatic vessels; externally, by tympanic membrane perforation; by direct extension from adjacent structures; from infection of the central nervous system; due to congenital infection (via the placenta); or during passage through the birth channel.

The diagnosis starts with a clinical history to identify contact with tuberculosis patients, previous treatment of this condition, and concomitant lesions, which may be found in 50% of cases, according to the literature.^8^, ^13^

The clinical picture is polymorphic and insidious, varying according to the immunological status of patients.^9^ The rule is: a patient with otorrhea and evidence of active tuberculosis in any other part of the body has tuberculosis of the ear until proven otherwise.^14^ The clinical features have changed over the years.^7^ It used to present as the triad: pain-free otorrhea, multiple tympanic membrane perforation, and peripheral facial palsy. Its current presentation has become polymorphic.^4^ Rather than pathognomonic features, there are specific findings, the most common of which are:^9^ significant otalgia, probably due to pressure caused by granulation tissue within the mastoid; serous otorrhea (mostly), which may become purulent due to secondary bacterial contamination;^4^, ^9^, ^12^ severe, early, sensorineural, mixed or conductive hearing loss in 90% of cases, which may persist after the infection has been completely treated, especially if therapy was initiated late.^1^, ^4^, ^11^, ^12^

Single or multiple tympanic perforation, denuded hammer of the ear, erosion of ossicles and even of the cortical bone of the mastoid, which may involve the bone capsule of the facial nerve, pallid granulation of the middle ear and mastoid cells, which may be mistaken for a cholesteatoma, and necrotic tissue, may all be seen on otoscopy. Granulation may eventually become hyperemic and friable.^1^, ^4^, ^8^, ^11^, ^14^, ^15^

There may be lymphadenomegaly of the neck and of the anterior and posterior regions to the ear on the physical examination.^8^, ^11^, ^14^

Complications occur mostly when the diagnosis is late, and include: peripheral facial paralysis, retroauricular fistulae, labyrinthitis, meningitis, tuberculous osteomyelitis of the petrous pyramid, subperiosteal, cerebral or cerebellar abscesses, acute mastoiditis and cellulites.^4^, ^11^, ^12^, ^13^ Peripheral facial palsy may be present in 15 to 40% of tuberculous otitis media cases, more frequently in children.4 The prognosis does not depend on decompression, but rather on early diagnosis and treatment. If the time interval between paralysis and the beginning of therapy is less than five days, complete recovery is expected; on the other hand, if this time frame is more than two months, there may be no recovery.^1^, ^4^, ^13^, ^14^ Intracranial invasion is rare, as the dura mater resists infection; if it occurs, it is usually secondary to blood borne dissemination.^1^, ^9^, ^11^, ^14^

An early diagnosis avoids surgery and complications; thus, this entity should be included in the differential diagnosis of all recalcitrant otites.^13^

Other chronically suppurative diseases that do not improve with conventional therapy should be considered in the differential diagnosis, such as: cholesteatoma, syphilis, Wegener's granulomatosis, fungal infection, eosinophyllic granulomatosis and sarcoidosis.^1^, ^4^, ^13^

Laboratory exams include:

Direct sputum bacilloscopy - should be a priority, as it allows the clinician to uncover the most important source of infection, the bacillus. It is important in cases of primary lung infection.

Bacteriology of ear secretions - not very reliable given the presence of other microorganisms that may interfere with the growth of Koch's bacillus and delay the diagnosis.^1^, ^9^, ^10^, ^13^ Chronic use of topical drops, such as neomycin, may alter the sensitivity of bacterial cultures.^13^, ^14^ The test is positive for bacilli in 20 to 30% of cases.^4^, ^6^, ^8^, ^15^ Whenever possible, drug susceptibility testing should be done after the microorganism is isolated, to identify resistant mycobacteria.^8^

Tuberculin test (PPD) - when positive, it suggests infection, but not necessarily tuberculous disease. This test may be difficult to interpret in BCG-vaccinated persons, since the vaccine may make the test positive. Test interpretation is as follows: 0-4mm, non-reacting, 5-9mm, weak reactor (TB-infected by atypical mycobacteria or BCG-vaccinated), 10mm or more, strong reactors (TB-infected, with disease or non-BCG vaccinated).

Histopathology of granulation tissue - when abundant, it is the most reliable diagnostic method; however, biopsies frequently need to be repeated for confirmation.^14^ Caseous necrosis and specific granulation with epithelioid cells and giant Langerhans cells may be seen.^4^, ^5^, ^9^, ^14^

Radiology - initially the tympanic cavity is filled, with no signs of bone erosion; later, ossicles may be destroyed, there may be sclerosis of the mastoid cortex, middle ear and mastoid cell opacification, increased density of the mastoid cortical bone and radioluscency if there is bone resorption.^4^, ^11^

The PCR genetic analysis - the results may be obtained from biopsy material within hours. It is useful for the early diagnosis, and is highly sensitive and specific for M. tuberculosis.^5^, ^6^, ^8^

DNA identification probe - uses the nuclei acid hybridization technique for identifying mycobacteria, yielding the result within hours. This technique is based on the association capability of nucleic acid complementary chains to associate and form a stable double-chain structure. The DNA probe is marked with a chemoluminescent marker that combines with the microorganism's ribosomal RNA to form a stable DNA:RNA hybrid.^6^

In pathology, mycobacterial infection may be seen to involve the connective tissue of the tympanic membrane, the middle ear mucosa, and the mastoid cells. It may reach the medullary area in the temporal bone. Histologically, the middle ear mucosa and the tympanic membrane may be inflamed and may contain giant cells, tubercles with Langerhans cells, calcification and ulcers. If inflammation is extensive, these tubercles may become necrotic and confluent. Bone is involved by continuity or inflammation. Erosion of the ossicles and Eustachian tube injury may occur in the middle ear. Bone destruction results in multiple bone sequestration. If untreated, granulomatous inflammation may progress towards the skin and regional lymph nodes, resulting in a usually retroauricular persistent fistula.^1^

Early therapy is paramount to avoid complications.^9^ Once it is started, there is rapid resolution of the infection.^13^, ^14^

In Brazil, three treatment regimens are recommended by the tuberculosis therapy guideline:^5^

The first regimen is recommended for the treatment of all forms of pulmonary or extrapulmonary tuberculosis, except for tuberculous meningitis or cases with associated HIV infection. Therapy last six months and is based on the drugs rifampicin, isoniazid and pyrazinamide.

The second regimen is indicated when the previous option has failed; in this case, therapy should last from 18 to 24 months. Recommended drugs, used in different combinations, are: isoniazid, rifampicin, pyrazinamide, ethambutol and streptomycin.

The third regimen was recommended by the Ministry of Health in 2000 for HIV patients, as follows: an initial regimen I (rifampicin, isoniazid and pyrazinamide during two months, followed by rifampicin and isoniazid during four months), which depends on CD4 T-cells and the HIV viral load. If HIV is associated with tuberculous meningoencephalitis, treatment last nine months (regimen II, with (rifampicin, isoniazid and pyrazinamide during two months, followed by rifampicin and isoniazid during seven months)^2^.

Surgery has a minor role but may be useful to provide polyp or granulation tissue for histology, and for treating complications.^2^, ^4^, ^11^, ^12^, ^15^,^16^ Surgery in aural tuberculosis aims to correct sequelae following medical treatment and cure of the disease.

## CASE REPORTS

Four cases of tuberculous otitis media are presented retrospectively. These cases were taken from three medical units in a 5-year period. Two of the patients were medical doctors, one was a chemical engineer and one case involved a minor in whose family there was active tuberculosis.

### Case 1

VCR, a female white patient aged 30 years, who was a medical doctor, presented fungal and bacterial external otitis that was treated initially with oral itraconazole and clindamycin and topical gentian violet followed by Burow's solution for 12 days. After therapy, two tympanic membrane perforations were noted, and fungal colonies reappeared. As suppuration continued, ear dressing with topical povidone in alternate days was recommended, and the patient was prescribed itraconazole and amoxicillin/clavulanic acid. One month later the patient presented left ear suppuration and pain. Treatment with amoxicillin and injectable steroids was done unsuccessfully; four days later, gatifloxacin was started. Seven days later the patient was reassessed because suppuration was still present, now with intense pain. Otoscopy revealed central perforation of the tympanic membrane, edema of the mucosa, a yellowish secretion and fungal colonies. The patient was treated with oral clindamycin, dressing for cleaning purposes and topical miconazole. Itraconazole per orum was started four days later. There was an initial improvement within 10 days, but the clinical picture eventually worsened. Further examinations were done because of this difficult progression. The chest X-ray was normal, computed tomography of the temporal bones showed that the middle ear was opacified and the mastoid was pneumatized and opacified. The complete blood count, HIV serology and syphilis serology were within normal limits or negative. As the treatment was unsuccessful and because a small bone sequestration was seen in the middle ear, with bone exposure in the promontory, tuberculosis was suspected. Culture for Koch's bacillus was negative for material biopsied from the ear. The 48-hour PPD measured 13mm. Based on these findings, triple therapy for tuberculosis was started with rifampicin (600mg/day for 6 months) plus isoniazid (400mg/day for 6 months) and pyrazinamide (2g/day for 15 days); topical isoconazole was also used. The clinical picture improved rapidly; the secretion and fungal infection regressed, granulation tissue developed, and the tympanic membrane started to heal. Twenty-five days later, the tympanic membrane had healed with slight retraction. Valsalva's maneuver showed that the tube was permeable.

One month later the patient reported improved hearing and less of the feeling of a blocked year. Six months later the tympanic membrane was healed, slightly atrophic and normal colored. Pure tone audiometry and voice investigation were within normal limits.

### Case 2

**Figure 1:**
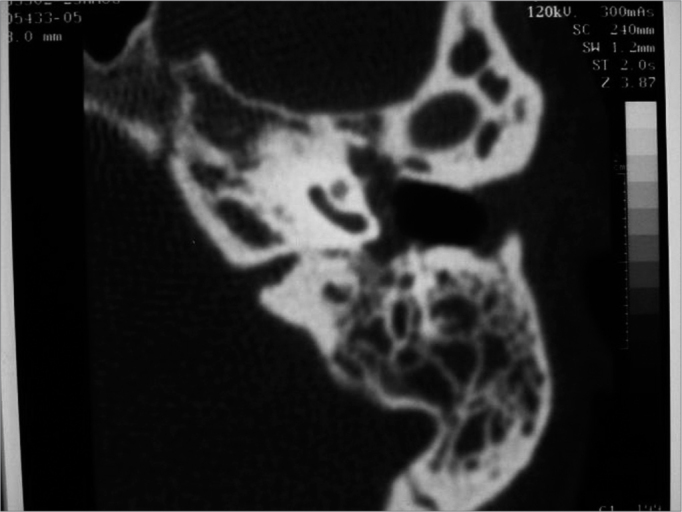
Case 2. Computed tomography showing treatment-resistant otomastoidits. Plain mastoidectomy was recommended.

**Figure 2:**
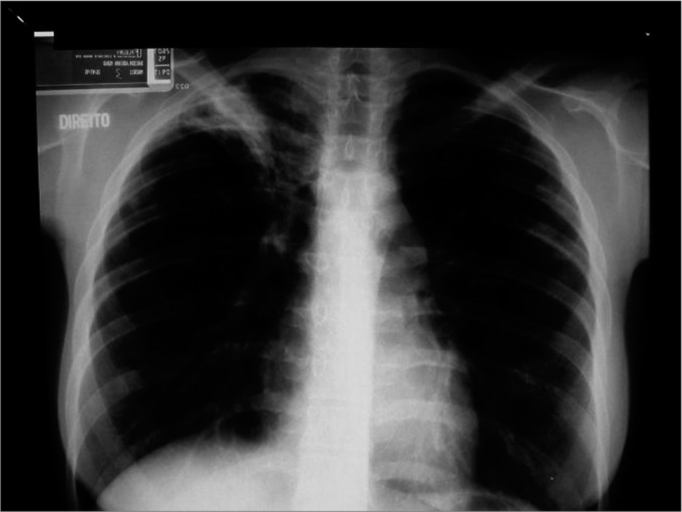
Case 2. Asymptomatic pulmonary involvement in the right apex and the left base of the lung.

AM, a white female patient aged 25 years, who was a medical doctor, complained of a blocked ear and no history of otitis. She sought a colleague for ear lavage. Three days later secretion started in that ear. The tympanic membrane was perforated. Therapy at this point included cephadroxil, followed by amoxicillin and clavulanate. The ear dried up but the tympanic membrane had four perforations. In the next four months, the same clinical picture reappeared and was treated with a variety of antibiotics. A computed tomography was done when the patient complained of dizziness (Figure 1), which showed that the mastoid and the inner ear were affected. A tympanomastoidectomy was indicated. The patient remained asymptomatic during the following four months, but hearing loss worsened. The ear was humid and adhesive otitis was noted. A ventilation tube was placed. As the ear was still secreting one month later, the patient was reexamined, which revealed that the epithelium of the tympanic membrane was thinned and that there was a clear secretion in the canal. Therapy was done using ciprofloxacin. One month later, the ventilation tube was eliminated; the tympanic membrane was perforated, through which it could be seen that the bone promontory was exposed and the long apophysis of the hammer was skeletized; the ear was humid, but with no suppuration. A chest X-ray (Figure 2) revealed typical pulmonary tuberculosis involving the right apex and the left base of the lung; the patient did not complain of coughing, weight loss or anorexia. The Mantoux test was highly positive and investigation of Koch's bacillus in sputum was negative. Therapy was done with rifampicin, isoniazid e pyrazinamide. Six months later there were still signs of activity, such as an ample tympanic membrane perforation, a humid promontory and exposure of the promontory bone and the external ear canal. Therapy was continued for nine months until signs of active disease had regressed; audiometry at this point revealed mixed hearing loss, mostly at low frequencies.

Tympanoplasty was done four years later; this procedure showed that the hammer was partially eroded and that the anvil was fixed. The anvil was removed, the head of the hammer was sectioned and the anvil was interposed between the rest of the hammer and the head of the stirrup. The tympanic membrane was repaired with a perichondral graft from the tragus. Six weeks later the perforation recurred close to the canal, and increased further by ten weeks. The myringoplasty was reviewed one year after the first procedure; the graft had a small perforation. The ear, however, was always dry, here was no vertigo or hearing loss.

### Case 3

**Figure 3:**
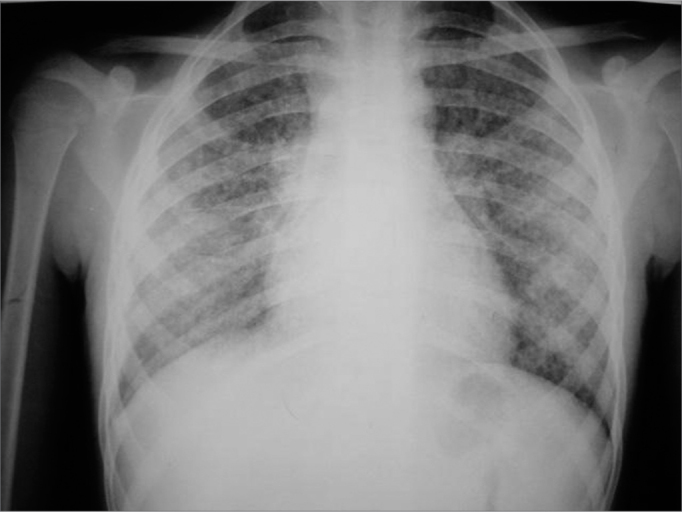
Case 3. Disseminated pulmonary reticulonodular infiltration.

PJCA, a black 10-year old male presented with continuous, bilateral otorrhea and no otalgia during the past four months, and that had not regressed with antibiotic therapy and ear protectors while bathing. The clinical picture was not associated with upper airway infection. The patient reported hypoacusis and rhinolalia since the beginning of symptoms. One month later the patient presented sporadic coughing with a whitish secretion and lost about 2 kg in weight. Fever was absent. The family history was exempt from infections. The patient constantly contacted a neighbor that was being treated for tuberculosis. Otoscopy revealed an abundant purulent secretion in both external ear canals; the bony portion of the anterior part of the ear canal was exposed and there was granulation on the posterior portion; abundant desquamation did not allow the tympanic membrane to be seen. Rhinoscopy showed purulent and bloody crusts in the nasal vestibules. The tonsils were edematous and covered by a whitish membrane; the uvula was edematous, the mucosa was granulomatous and hyperemic and there was pus in the nasopharynx. Inspection revealed enlarged cervical lymph nodes; lymph nodes of all the head and neck chains were enlarged, softened, mobile and painless, measuring about 2.0 to 2.5 cm. Lung auscultation showed decreased sounds and rhonchi in the lower portion of the right lung. The chest X-ray (Figure 3) revealed a diffuse reticular-nodular infiltrate. The patient was admitted into hospital with fever, otorrhea and dyspnea. The pediatrician prescribed clindamycin while laboratory test were undertaken. Computed tomography revealed soft tissue density material within the middle ear and mucosal thickening in the maxillary sinuses. Computed tomography of the lung showed multiple micronodules throughout the parenchyma that coalesced in the apices of both lungs; the suggested diagnosis was miliary tuberculosis. Rigid telescopy of the larynx revealed an infiltrated ulcer of the left arytenoid and base of the epiglottis, and mobile vocal folds. The complete blood count showed anemia. Three sputum studies for Koch's bacillus were negative. HIV serology was non-reacting. The T lymphocyte count was as follows: CD4 - 698 cells/microliter, CD8 - 285 cells/microliter and CD4/CD8: 2.45. The hemosedimentation rate was: 79 mm/h. The PPD was negative. Culture of the ear secretion revealed Staphylococcus sp. Pathology studies of the granulation tissue in the ear canal showed a chronic inflammation that suggested tuberculosis.

The patient was treated with a triple regimen (isoniazid, rifampicin and pyrazinamide). Improvement of the clinical picture allowed the patient to be discharged seven days later; only the external auditory canal was stenotic. At this point audiometry showed moderate conductive hearing loss in the left ear.

### Case 4

DA, a white female patient aged 35 years, who was a chemical engineer, arrived at our unit after 50 days of treatment for otomycosis. Topical ketoconazole, tolciclate, polymyxin B sulfate and neomycin were attempted, together with ciprofloxacin, metronidazole and sulphametoxazol/trimethoprim, without improvement. The first examination revealed a suppurating ear with posterior perforation of the tympanic membrane, which was necrotic throughout. The patient was medicated with topical and oral ciprofloxacin. One week later there was a fungal colony in the canal and over the hammer. The colony was removed, and necrosis was noted in part of the hammer and the external auditory canal. The patient reported pain over the mastoid; thus, a biopsy was made of the necrotic material and the patient was treated with oral fluconazole and six injections of ceftriaxone. Pathology showed a chronic inflammation with ulcers, suppuration and fungi. A swab of the secretion revealed no alcohol-acid resistant bacilli. Computed tomography of the temporal bone revealed opacification of the mastoid cells with no bone erosion. One month later there was still suppuration, the tympanic membrane was absent, part of the hammer was eroded and the promontory had granulation tissue and a fungal colony. Audiometry showed severe mixed hearing loss at low frequencies and moderate hearing loss at middle frequencies. The chest X-ray was suggestive of tuberculosis. The patient was referred to a pneumologist who started therapy with isoniazid, rifampicin and flixotide during six months. One year later, the patient was discharged from the medical treatment. The ears had no secretions, there was hearing loss, full loss of the tympanic membrane and partial loss of the ossicles. Tympanoplasty was undertaken after one year of no active disease. The head of the hammer was removed and the anvil was interposed over the head of the stirrup; a perichondral graft was used for rebuilding the membrane. The patient was reoperated six months later to close to minor marginal perforations. The patient was finally discharged from medical therapy eight months later; the tympanic membrane was complete and there was mixed moderate hearing loss at low frequencies and mild loss at high frequencies.

Monitoring of the lungs was done for three years, and showed no further activity of the disease.

## DISCUSSION

It is interesting to note that in these four cases, two of them (50%) were young and previously healthy medical doctors. It might, therefore, be considered a profession-related disease, although this has not been reported in the literature. These cases are an important warning for medical doctors who have any contact with tuberculous patients. Even young, healthy professionals, with apparently intact immune systems, should be aware of the risk and should take measures to avoid contamination.

None of these patients had any immunosuppressing diseases, such as HIV infection or diabetes. One patient worked in an environment that was harmful to upper airways; thus, any individual may be susceptible to tuberculosis if there is contact with patients that have this disease.

These cases show that it may be difficult to demonstrate tuberculosis of the ear, even when the possibility of this disease is raised; at times, only the response to therapy confirms the etiology. Although tuberculosis of the ear is rare, otologists should diagnose this condition as early as possible to avoid unnecessary treatments and sequelae. Physicians should bear in mind that that this condition may manifest initially as apparently routine otomycosis or otitis media, but which does not improve with therapy. The classical sign of single or multiple tympanic perforations with no intense acute otitis media should be remembered.

The important features of these cases are the insidious disease progression, the presence of recurring otomycosis and infection that resist usual therapy, necrosis of soft tissues in the external and middle ear, bone exposure (especially on the promontory, but also of the canal) and erosion of the ossicles.

Poor results of corrective surgery, seen in cases two and four where the bone was exposed, are probably due to inadequate blood circulation of scarring tissues on the graft bed. Surgeons should be aware of the need to inform patients about the poor prognosis of surgical reconstruction in such cases.

## FINAL COMMENTS

Contamination of healthy individuals may occur; healthcare workers should be especially attentive. Tuberculosis should be included as a differential diagnosis in cases of recalcitrant otomycosis and otitis media. Classical signs of ear tuberculosis remain valid, albeit not always remembered. In the study cases, the clinical picture, chest X-rays and the PPD were the most sensitive diagnostic methods for this condition. Grafts in ears with sequelae of tuberculosis do not take readily. Tuberculosis of the ear is highly sensitive to standard medical therapy for pulmonary tuberculosis; thus, treatment should be started promptly to avoid sequelae.
